# Dietary magnesium intake is protective in patients with periodontitis

**DOI:** 10.3389/fnut.2022.976518

**Published:** 2022-08-25

**Authors:** Xin-yu Li, Ming-zhe Wen, Hui Liu, Yu-chen Shen, Li-xin Su, Xi-tao Yang

**Affiliations:** ^1^Department of Interventional Therapy, Multidisciplinary Team of Vascular Anomalies, Shanghai Ninth People's Hospital, Shanghai Jiao Tong University, Shanghai, China; ^2^Department of Neurosurgery, Shanghai Ninth People's Hospital, Shanghai JiaoTong University School of Medicine, Shanghai, China; ^3^Department of Nephrology, Shanghai Jiao Tong University Affiliated Sixth People's Hospital, Shanghai, China

**Keywords:** periodontal disease, magnesium, periodontitis, dietary structure, Mg

## Abstract

**Background:**

Periodontitis is a chronic inflammatory disease of the oral cavity characterized by inflammation of the periodontal tissue and resorption of the alveolar bone, which has a high incidence and is the main cause of tooth loss in adults. In addition to its role in promoting osteogenesis, magnesium also has a role in regulating the inflammatory response, both systemically and locally. There is growing evidence that magnesium is an important factor in maintaining the normal functioning of the body's immune system. Hypomagnesaemia can lead to a variety of chronic inflammatory diseases throughout the body, including periodontitis. Two-thirds of the US population suffers from magnesium deficiency. The connection between dietary magnesium and periodontitis is unknown. As a result, we set out to investigate the link between dietary magnesium intake and periodontitis.

**Methods:**

In this study, we collected data from the National Health and Nutrition Examination Survey (NHANES) database from 2013 to 2014. Through 24-h dietary recalls, information about food consumption was collected. We examined the association between the dietary magnesium and periodontitis using multivariable logistic regression model. Based on odds ratios (OR) and 95% confidence intervals (CIs), a strong association was detected.

**Results:**

Multivariable logistic regression analysis showed that the OR for periodontitis comparing the highest to the lowest quintile of dietary magnesium intake was 0.69 (95% CIs = 0.52~0.92). The restricted cubic spline (RCS) analysis showed that the non-linear association between dietary magnesium and periodontitis was statistically significant and that dietary magnesium supplementation reduced the prevalence of periodontitis.

**Conclusion:**

Dietary magnesium intake is associated with the prevalence of periodontitis. Dietary magnesium deficiency increases the prevalence of periodontitis.

## Introduction

Periodontitis is a highly prevalent chronic, oral multi-bacterial infection disease that affects nearly half of the population worldwide (1). Periodontitis is not only a localized inflammatory disease of the oral cavity, but there is increasing evidence that periodontitis is closely associated with the development of systemic diseases such as cardiovascular disease, diabetes, Parkinson's disease and infective endocarditis (2–7). The prevalence of periodontitis in China is over 50%, and among people aged 60–74 years, the prevalence is as high as 70–90% (8). Furthermore, micronutrients also play a crucial role in periodontitis. Studies have shown that periodontitis causes changes in the levels of some micronutrients in the body and that imbalances in vitamins and minerals in the body have a significant impact on the development of periodontal disease (9). Increased dietary magnesium intake may prevent the incidence of diabetes, metabolic syndrome, hypertension and cardiovascular disease (10). Previous studies have found that vitamins A, B, C, calcium, zinc and polyphenols can prevent periodontitis, while the role of magnesium in periodontitis remains unclear. Given the increasing prevalence of periodontitis and the low dietary intake of magnesium, it is critical to determine whether magnesium intake is associated with periodontitis prevalence. The goal of this study was to determine the relationship between dietary magnesium and periodontitis.

## Methods

### Study population

We analyzed retrospective NHANES 2013-2014 data. In addition to a home interview, NHANES participants received a physical examination and interviews at a mobile examination center (MEC) (11). Patients must be at least 30 years of age, have at least one natural tooth (excluding third molars), and not be suffering from any condition that requires antibiotic prophylaxis before periodontal probing in order to qualify for a full-mouth periodontal examination (12). Because only a subset of NHANES participants underwent MEC examinations, we included only those who reported a complete dental examination. In the first 24-h recall, trained interviewers collected food recalls in person with the automated multiple-pass technique used by the United States Department of Agriculture (USDA). We also included other demographic variables (including age, gender, race, educational attainment, smoking status, and alcohol use status) and BMI (Body Mass Index). Participants with missing data were excluded. Ultimately, a total of 3,028 participants were included in the final analyses.

### Study variables

#### Socio-demographic characteristics

The NHANES collected self-reported information about age, gender, race, education, smoking status, and alcohol consumption. Sociodemographic characteristics were set as age, gender (male/female), race (Mexican American; white; black and other), education level (below high school; high school and college or above), smoking status (former; never and current), drinking status (never; former; light; moderate and heavy) and poverty income ratio (PIR). NHANES recorded the participants' smoking status, duration and smoking-related behaviors (13). The classification of smoking status is based on the following criteria (14): Never smokers were defined as adults who had never smoked or smoked <100 cigarettes in their lifetime; smokers who reported smoking ≥100 cigarettes in their lifetime and were currently non-smokers were identified as former smokers; and current smokers were defined as smokers who smoked ≥100 cigarettes on certain days or days in their lifetime. Never drinkers were defined as those who reported drinking <12 drinks; ever drinkers were defined as those who had more than 12 drinks in their lifetime but not in the past year; current drinkers were further classified as light, moderate and heavy current drinkers. Heavy drinkers were defined as ≥3 drinks per day for women and ≥4 drinks per day for men, with 5 or more binge drinking days per month; moderate drinkers were defined as ≥2 drinks per day for women and ≥3 drinks per day for men, with ≥2 binge drinking days per month. Light drinkers: did not meet the above criteria (15, 16). PIR measures the relationship between family income and poverty threshold to reflect socioeconomic status: low (PIR < 1.35), medium (1.35 ≤ PIR < 3.0) and high (PIR ≥ 3.0) (17, 18). Using the WHO classification, there are four categories of BMI: Underweight is <18.5, normal weight = 18.5–24.9, overweight is 25–29.9, and obese is >30.0 (19).

#### Periodontitis

Each participant will go through a periodontal clinical examination by one calibrated examiner to assess his or her periodontal health (20). The periodontal examination includes probing depth (PD) and clinical attachment level (AL) of the MEC. Supplementary Table 1 shows the classification criteria according to periodontal status.

### Dietary magnesium intake

Face-to-face 24-h food memories collected during the MEC visit as part of the USDA's “What We Eat in America.” Dietary recall notices were sent to customers in person by trained staff using a USDA Automated Multiple-Pass Method (21). In addition, information was collected on the type, frequency of consumption, duration and amount taken of each reported dietary supplement. The average daily intake of magnesium from dietary supplements was calculated for participants using the reported number of days of supplement use, the reported daily dose and the serving size unit on the product label. Total magnesium intake was calculated as the sum of dietary and supplement intake (22).

### Statistical analysis

For descriptive analyses, categorical variables were described using frequencies and percentages, and continuous variables were described using means and standard deviations. We stratified the cohort according to quintiles of magnesium intake daily in the primary analysis. To evaluate differences in distribution of categorical variables, Fisher's exact test was used (23). We then present descriptive statistics comparing the prevalence of periodontitis with dietary magnesium. We examined the relationship between dietary magnesium intake and periodontitis using univariate and multivariate logistic regression models, adjusting for age, sex, race, smoking, alcohol consumption, education level and BMI.

## Results

### Baseline characteristics

**Figure 1:**
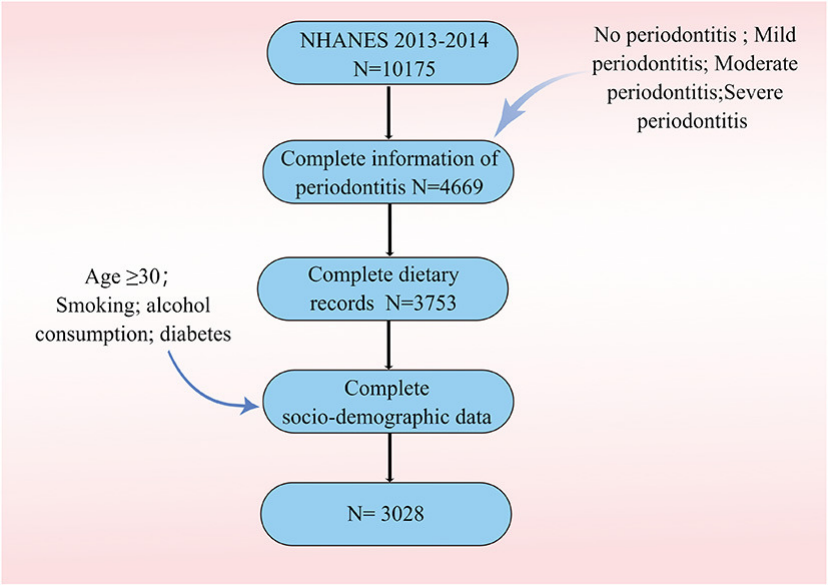
The overview of the flow chart for this paper.

Figure 1 describes study recruitment and inclusion/exclusion criteria. Table 1 displays the subjects' baseline characteristics. Compared to those in the top quintile of magnesium intake, those with lower dietary magnesium intake had a lower educational attainment, lower income, higher average age, higher rates of smoking, higher rates of previous and moderate alcohol consumption, higher prevalence of diabetes and a greater likelihood of obesity. Baseline periodontitis, age, sex, race, PIR, education, smoke, alcohol, DM, BMI were different between groups (*P* < 0.05).

**Table 1:** Characteristics of the overall target population according to dietary magnesium intake.

**Variables**	**Total** **(*n* = 3,028)**	**Q1** **(*n* = 602)**	**Q2** **(*n* = 607)**	**Q3** **(*n* = 606)**	**Q4** **(*n* = 607)**	**Q5** **(*n* = 606)**	***P***
Periodontitis, n (%)							0.001
No	1,748 (57.7)	304 (50.5)	357 (58.8)	368 (60.7)	351 (57.8)	368 (60.7)	
Yes	1,280 (42.3)	298 (49.5)	250 (41.2)	238 (39.3)	256 (42.2)	238 (39.3)	
Periodontitis Severity *n* (%)							0.03
Mild	37 (1.2)	11 (1.8)	5 (0.8)	8 (1.3)	8 (1.3)	5 (0.8)	
Moderate	1,001 (33.1)	230 (38.2)	200 (32.9)	190 (31.4)	191 (31.5)	190 (31.4)	
No	1,748 (57.7)	304 (50.5)	357 (58.8)	368 (60.7)	351 (57.8)	368 (60.7)	
Severe	242 (8.0)	57 (9.5)	45 (7.4)	40 (6.6)	57 (9.4)	43 (7.1)	
Age, mean ± SD	52.2 ± 14.2	53.8 ± 14.5	52.2 ± 14.3	52.1 ± 14.5	52.2 ± 14.1	50.6 ± 13.3	0.003
Sex, *n* (%)							<0.001
Female	1,592 (52.6)	426 (70.8)	376 (61.9)	323 (53.3)	281 (46.3)	186 (30.7)	
Male	1,436 (47.4)	176 (29.2)	231 (38.1)	283 (46.7)	326 (53.7)	420 (69.3)	
Race, *n* (%)							<0.001
Mexican American	396 (13.1)	52 (8.6)	69 (11.4)	91 (15)	85 (14)	99 (16.3)	
Non-hispanic black	589 (19.5)	171 (28.4)	137 (22.6)	116 (19.1)	84 (13.8)	81 (13.4)	
Non-hispanic white	1,365 (45.1)	272 (45.2)	267 (44)	266 (43.9)	299 (49.3)	261 (43.1)	
Other race	678 (22.4)	107 (17.8)	134 (22.1)	133 (21.9)	139 (22.9)	165 (27.2)	
PIR, *n* (%)							<0.001
High	1,254 (44.6)	167 (29.7)	239 (41.9)	274 (49.2)	270 (49)	304 (53.2)	
Low	835 (29.7)	237 (42.2)	181 (31.8)	149 (26.8)	138 (25)	130 (22.8)	
Medium	722 (25.7)	158 (28.1)	150 (26.3)	134 (24.1)	143 (26)	137 (24)	
Education, *n* (%)							<0.001
9–11th grade (includes 12th grade with no diploma)	340 (11.2)	95 (15.8)	81 (13.3)	59 (9.7)	56 (9.2)	49 (8.1)	
College graduate or above	897 (29.6)	96 (16)	150 (24.7)	192 (31.7)	214 (35.3)	245 (40.4)	
High school graduate/GED or equivalent	672 (22.2)	157 (26.1)	146 (24.1)	130 (21.5)	137 (22.6)	102 (16.8)	
<9th grade	187 (6.2)	40 (6.7)	32 (5.3)	42 (6.9)	39 (6.4)	34 (5.6)	
Some college or AA degree	931 (30.8)	213 (35.4)	198 (32.6)	183 (30.2)	161 (26.5)	176 (29)	
Smoke, *n* (%)							<0.001
Former	768 (25.4)	147 (24.5)	148 (24.4)	133 (21.9)	161 (26.5)	179 (29.5)	
Never	1,740 (57.5)	313 (52.2)	355 (58.5)	374 (61.7)	355 (58.5)	343 (56.6)	
Now	518 (17.1)	140 (23.3)	104 (17.1)	99 (16.3)	91 (15)	84 (13.9)	
Alcohol, *n* (%)							<0.001
Former	494 (16.8)	118 (20.2)	102 (17.3)	93 (15.9)	98 (16.6)	83 (14.1)	
Heavy	483 (16.4)	92 (15.8)	76 (12.9)	101 (17.3)	101 (17.1)	113 (19.2)	
Mild	1,098 (37.4)	177 (30.4)	215 (36.5)	229 (39.2)	226 (38.2)	251 (42.5)	
Moderate	454 (15.5)	97 (16.6)	100 (17)	89 (15.2)	95 (16.1)	73 (12.4)	
Never	408 (13.9)	99 (17)	96 (16.3)	72 (12.3)	71 (12)	70 (11.9)	
DM, *n* (%)							<0.001
DM	432 (14.3)	104 (17.3)	107 (17.9)	69 (11.4)	90 (14.9)	62 (10.3)	
No	2,579 (85.7)	496 (82.7)	491 (82.1)	534 (88.6)	516 (85.1)	542 (89.7)	
BMI, *n* (%)							<0.001
Normal weight	751 (24.9)	125 (21)	133 (22)	155 (25.6)	156 (25.8)	182 (30.1)	
Obese	1,221 (40.5)	289 (48.5)	273 (45.2)	228 (37.6)	236 (39.1)	195 (32.2)	
Overweight	1,012 (33.6)	173 (29)	193 (32)	217 (35.8)	205 (33.9)	224 (37)	
underweight	31 (1.0)	9 (1.5)	5 (0.8)	6 (1)	7 (1.2)	4 (0.7)	

### Outcome and exposure factors

We categorized the participants into quintiles based on the daily magnesium consumption and periodontitis ever accounted for 49.5% in Q1, 41.2% in Q2, 39.3% in Q3, 42.2% in Q4 and 39.3% in Q5. Participants in the top quintile of dietary magnesium intake had a lower prevalence of periodontitis than those in the bottom quintile. In addition, in terms of severity of periodontitis, mild, moderate and severe periodontitis were both more prevalent in the group of participants with the lowest dietary intake of magnesium.

### Multivariate regression analysis

Based on quintiles of dietary magnesium intake, all the models we developed showed a negative association between dietary magnesium intake and the prevalence of periodontitis (Table 2). That is, logistic regression analysis showed that the highest quintile of dietary magnesium intake was associated with a lower prevalence of periodontitis. Compared to the lowest quintile of the dietary magnesium intake, the top quintile had a lower prevalence of periodontitis in model 1 (OR =0.65; 95%CI 0.5~0.86), model 2 (OR= 0.69; 95%CI 0.52~0.92) and model 3 (OR = 0.69; 95%CI 0.52~0.92). P for trend was < 0.05 in all models.

**Table 2:** Association of dietary magnesium intake with periodontitis.

**Exposure**	**Unadjusted model**	**Model 1**	**Model 2**	**Model 3**
Mg _Q1	1 (Ref)	1 (Ref)	1 (Ref)	1 (Ref)
Mg _Q2	0.71 (0.57~0.9)	0.78 (0.6~1.01)	0.8 (0.61~1.04)	0.79 (0.61~1.03)
Mg _Q3	0.66 (0.53~0.83)	0.72 (0.56~0.94)	0.74 (0.57~0.98)	0.76 (0.58~1)
Mg _Q4	0.74 (0.59~0.93)	0.85 (0.66~1.11)	0.9 (0.68~1.18)	0.88 (0.67~1.16)
Mg_Q5	0.66 (0.53~0.83)	0.65 (0.5~0.86)	0.69 (0.52~0.92)	0.69 (0.52~0.92)
*P*-value for trend	0.002	<0.001	0.02	0.03

Model 1: adjusted for gender, age, race.

Model 2: Model 1+ adjusted for smoke, PIR, alcohol.

Model 3: Model 2+smoke, alcohol and BMI, DM.

### Dose-response relationship between dietary magnesium intake and periodontitis

**Figure 2:**
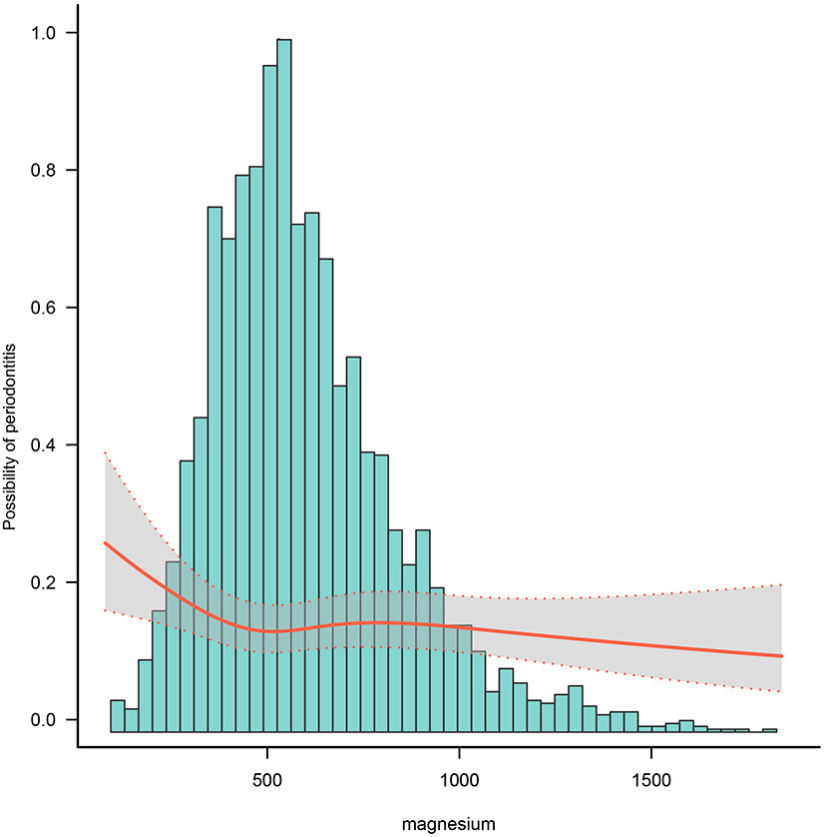
Dose-response relationship of dietary magnesium with periodontitis.

After adjusting for potential confounders, RCS analysis showed indicated a non-linear relationship between dietary magnesium intake and periodontitis (Figure 2). As can be seen from the diagram, the slope is steeper in the first 1/3 of the line, and the subsequent line remains on a downward trend. It is suggested that dietary magnesium intake is negatively associated with the prevalence of periodontitis.

### Subgroup analyses

**Figure 3:**
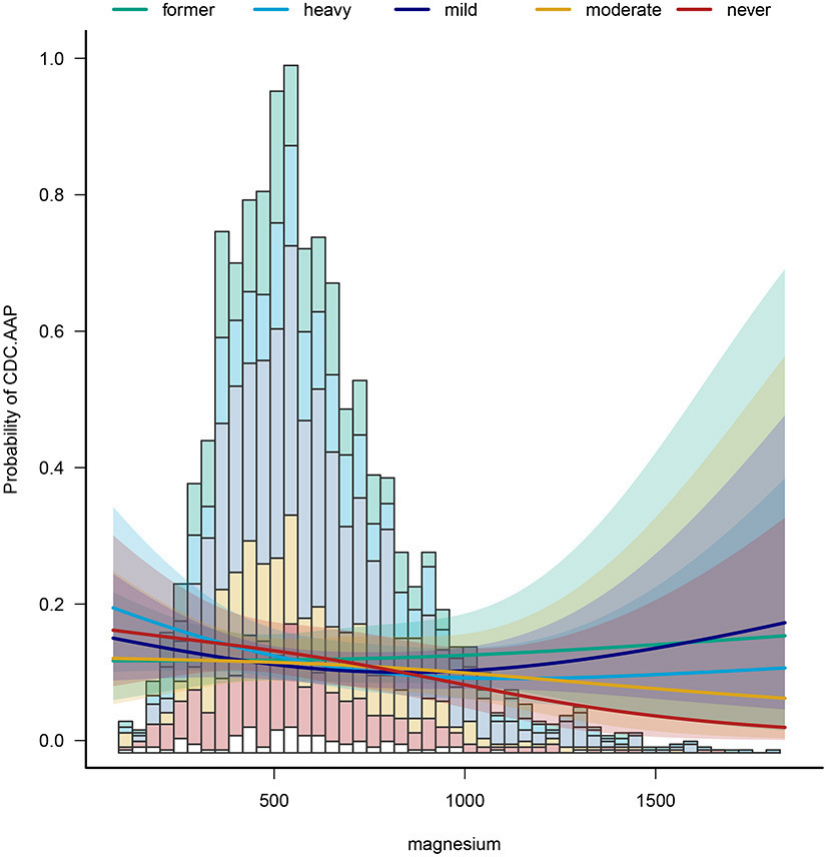
The relationship between dietary magnesium intake on the prevalence of periodontitis in participants with different drinkers.

To identify potential effect modifiers, we also conducted a subgroup analysis (Table 3). The results showed that the effect size of the relationship was significantly different across sex, education level and across alcohol. As for the sex, in the group of women with the highest dietary magnesium intake, there was a 40% reduction in the prevalence of periodontitis. In men, all five dietary magnesium intakes had a protective effect, with a 41% reduction in the prevalence of periodontitis in the most protective group. Also, we found that for heavy drinkers, increasing dietary magnesium intake reduced the prevalence of periodontitis by 54%. In mild drinkers, a 40% reduction in periodontitis intake was observed. Figure 3 shows the relationship between dietary magnesium intake on the prevalence of periodontitis in participants with different drinkers.

**Table 3:** Stratifiedlogistic regression analysis to identify variables that modify the correlation.

**Subgroup**	**Variable**	**OR_95CI**	***P*_value**	**P.for.interaction**
Age ≤ 60				0.523
	MgQ1			
	MgQ2	0.82 (0.58~1.16)	0.265	
	MgQ3	0.88 (0.62~1.24)	0.463	
	MgQ4	0.89 (0.62~1.26)	0.499	
	MgQ5	0.69 (0.49~0.99)	0.043	
Age >60				
	MgQ1	1 (Ref)		
	MgQ2	0.76 (0.49~1.18)	0.215	
	MgQ3	0.58 (0.36~0.93)	0.024	
	MgQ4	0.88 (0.55~1.39)	0.578	
	MgQ5	0.7 (0.43~1.15)	0.16	
Sex = female				**0.042**
	MgQ1	1 (Ref)		
	MgQ2	0.91 (0.65~1.27)	0.563	
	MgQ3	0.86 (0.6~1.23)	0.415	
	MgQ4	1.25 (0.86~1.81)	0.247	
	MgQ5	0.6 (0.39~0.95)	0.028	
Sex = male				
	MgQ1	1(Ref)		
	MgQ2	0.6 (0.37~0.95)	0.031	
	MgQ3	0.59 (0.37~0.94)	0.025	
	MgQ4	0.6 (0.38~0.93)	0.022	
	MgQ5	0.61 (0.4~0.94)	0.026	
Less than high school				**0.038**
	MgQ1	1 (Ref)		
	MgQ2	0.36 (0.19~0.66)	0.001	
	MgQ3	0.69 (0.35~1.34)	0.267	
	MgQ4	0.61 (0.31~1.21)	0.159	
	MgQ5	0.69 (0.32~1.46)	0.327	
High school				
	MgQ1	1 (Ref)		
	MgQ2	0.79 (0.46~1.35)	0.381	
	MgQ3	0.92 (0.52~1.63)	0.784	
	MgQ4	0.89 (0.5~1.55)	0.67	
	MgQ5	0.77 (0.41~1.46)	0.423	
College or above				
	MgQ1	1 (Ref)		
	MgQ2	1.07 (0.74~1.55)	0.734	
	MgQ3	0.75 (0.51~1.09)	0.132	
	MgQ4	0.99 (0.68~1.45)	0.971	
	MgQ5	0.77 (0.53~1.12)	0.167	
Smoke = former				0.163
	MgQ1	1 (Ref)		
	MgQ2	1 (0.59~1.71)	0.987	
	MgQ3	0.73 (0.42~1.29)	0.282	
	MgQ4	1.08 (0.64~1.85)	0.765	
	MgQ5	0.85 (0.5~1.46)	0.558	
Smoke = never				
	MgQ1	1 (Ref)		
	MgQ2	0.83 (0.58~1.21)	0.34	
	MgQ3	0.69 (0.47~1.01)	0.056	
	MgQ4	0.83 (0.56~1.22)	0.343	
	MgQ5	0.69 (0.46~1.03)	0.067	
Smoke = now				
	MgQ1	1 (Ref)		
	MgQ2	0.44 (0.24~0.84)	0.012	
	MgQ3	0.97 (0.5~1.89)	0.935	
	MgQ4	0.59 (0.3~1.19)	0.142	
	MgQ5	0.46 (0.23~0.94)	0.033	
Alcohol.user = former				**0.024**
	MgQ1	1 (Ref)		
	MgQ2	0.94 (0.49~1.79)	0.848	
	MgQ3	1 (0.51~1.96)	0.998	
	MgQ4	1.44 (0.74~2.8)	0.285	
	MgQ5	1.14 (0.58~2.26)	0.705	
Alcohol.user = heavy				
	MgQ1	1 (Ref)		
	MgQ2	0.53 (0.25~1.1)	0.088	
	MgQ3	0.81 (0.4~1.62)	0.551	
	MgQ4	0.46 (0.23~0.93)	0.031	
	MgQ5	0.61 (0.3~1.22)	0.159	
Alcohol.user = mild				
	MgQ1	1 (Ref)		
	MgQ2	0.57 (0.35~0.92)	0.022	
	MgQ3	0.71 (0.44~1.15)	0.17	
	MgQ4	0.96 (0.59~1.55)	0.858	
	MgQ5	0.6 (0.37~0.99)	0.044	
Alcohol.user = moderate				
	MgQ1	1 (Ref)		
	MgQ2	1.42 (0.72~2.8)	0.305	
	MgQ3	0.47 (0.22~1.01)	0.052	
	MgQ4	0.86 (0.42~1.75)	0.679	
	MgQ5	0.91 (0.42~1.98)	0.819	
Alcohol.user = never				
	MgQ1	1 (Ref)		
	MgQ2	0.96 (0.49~1.9)	0.917	
	MgQ3	0.65 (0.31~1.38)	0.266	
	MgQ4	0.76 (0.35~1.69)	0.506	
	MgQ5	0.35 (0.14~0.84)	0.019	
DM = DM				0.665
	MgQ1	1 (Ref)		
	MgQ2	0.83 (0.45~1.54)	0.562	
	MgQ3	0.65 (0.32~1.31)	0.23	
	MgQ4	1.07 (0.54~2.11)	0.849	
	MgQ5	1.02 (0.47~2.2)	0.957	
DM = no				
	MgQ1	1 (Ref)		
	MgQ2	0.77 (0.57~1.04)	0.091	
	MgQ3	0.78 (0.58~1.05)	0.103	
	MgQ4	0.85 (0.63~1.16)	0.303	
	MgQ5	0.65 (0.48~0.88)	0.006	

Bold values indicate significant P-values (<0.05).

## Discussion

This cross-sectional study examined the association of dietary magnesium intake and periodontitis.

Our analysis showed that a higher dietary magnesium intake was significantly associated with a lower prevalence of periodontitis. Multivariate logistic regression analysis revealed an OR for periodontitis of 0.69 (95% CIs = 0.52–0.92) for the highest quintile of dietary magnesium intake vs. the lowest quintile.

Magnesium is an essential nutrient that is required for a variety of physiologic functions in the body. The consequences of magnesium deficiency go beyond oral lesions and may have an impact on the course of the COVID-19 pandemic (24). Most of the magnesium absorbed by the body comes from foods such as nuts, seeds, whole grains and green leafy vegetables, while magnesium is necessary for the maintenance and formation of calcified tissues such as bone (25, 26). Studies have shown a complex internal relationship between periodontitis and diabetes (27). In obese men, the risk of periodontitis is significantly higher in those on a high-fat diet than in those on a healthy diet (27). Shimabukuro's study concluded that toothpastes containing magnesium salts could reduce the incidence of periodontitis (28). Staudte et al. recorded the food intake of 42 patients with periodontitis for 1 week and compared it with 38 healthy subjects. The results of the study concluded that dietary deficiency of magnesium has a negative impact on periodontal health (29).

Of the people included in our current study, 42.3% had periodontitis. We found that the prevalence of periodontitis was higher in older men over 55 years of age, Non-Hispanic White, low income groups, high school education and below, with a history of smoking and obesity. In addition, we found that the prevalence of periodontitis was more prevalent in participants with reduced dietary magnesium intake. After adjusting for gender, age, race, BMI, poverty, alcohol consumption, diabetes and smoking status, the results showed that the group with the highest dietary magnesium intake was less likely to develop periodontal disease compared to the group of participants with the lowest dietary magnesium intake. These results suggest that a lack of magnesium in the daily diet increases the prevalence of periodontal disease. Our results are also in agreement with the findings of Shimabukuro and Staudte et al. above (25, 26). In the UK, the recommended intake of magnesium is 300 mg/day for men and 270 mg/day for women (30). Our analysis found the most significant protective effect on periodontitis for dietary magnesium intake in the range up to 500 mg, with a subsequent negative correlation remaining as dietary magnesium intake increased, but the protective effect was diminishing. This suggests that adjusting dietary magnesium intake to the recommended dose may have a preventive effect on periodontitis. Our findings support previous research that magnesium supplementation reduces the incidence of periodontitis, prevents tooth loss in middle-aged people, and delays tooth loss in the elderly. As a result, the individual's wellbeing will improve, and the cost of treatment and restoration will be reduced (31).

In typical Western diets, saturated fats, sucrose, and fructose, proteins from red meat, and sodium are in high quantities, while monounsaturated and polyunsaturated fats are in low amounts (32). The Mediterranean or modern DASH (Dietary Approaches to Stop Hypertension) diet is rich in vegetables and fruit, whole grains, low-fat dairy products, poultry, fish, nuts, legumes and seeds, and is also rich in magnesium, calcium, potassium and fiber, plus low in trans fatty acids, saturated acids and cholesterol. In previous studies the DASH diet and the Mediterranean diet have been associated with a reduced risk of cardiovascular disease, stroke, heart failure, diabetes and cancer. Since periodontitis is potentially associated with cardiovascular disease, avoiding the Western diet and adopting the DASH and Mediterranean diets may not only meet dietary magnesium intake but also reduce the prevalence of periodontitis. Based on the data of NHANES, this study explored the relationship between dietary magnesium intake and periodontitis, and further analyzed the dose-response relationship. There are still a few limitations in this study. As a result of information bias, especially recall bias, questionnaires often lead to inaccurate exposure estimations. Second, parallel adjustment for strongly correlated nutrients may not be the best way to identify the impact of a single nutrient independently, as the regression coefficients and standard errors tend to be unstable as covariance rises, making interpretation challenging. Finally, we cannot exclude the possibility of other unknown confounders.

## Conclusion

Our study of the NHANES database found that dietary magnesium intake was negatively associated with the incidence of periodontitis, that two-thirds of the US population has magnesium deficiency, and that increasing magnesium intake through dietary modification could help reduce the prevalence of periodontitis.

## Data availability statement

The original contributions presented in the study are included in the article/Supplementary material, further inquiries can be directed to the corresponding authors.

## Author contributions

All authors made substantial contributions to conception and design, acquisition of data, or analysis and interpretation of data, took part in drafting the article or revising it critically for important intellectual content, agreed to submit to the current journal, gave final approval of the version to be published, and agree to be accountable for all aspects of the work.

## Conflict of interest

The authors declare that the research was conducted in the absence of any commercial or financial relationships that could be construed as a potential conflict of interest.

## Publisher's note

All claims expressed in this article are solely those of the authors and do not necessarily represent those of their affiliated organizations, or those of the publisher, the editors and the reviewers. Any product that may be evaluated in this article, or claim that may be made by its manufacturer, is not guaranteed or endorsed by the publisher.
